# Variance components linkage analysis for adjusted systolic blood pressure in the Framingham Heart Study

**DOI:** 10.1186/1471-2156-4-S1-S4

**Published:** 2003-12-31

**Authors:** Martyn C Byng, Sheila A Fisher, Cathryn M Lewis, John C Whittaker

**Affiliations:** 1 Division of Genetics and Development, Guy's, King's and St. Thomas' School of Medicine, 8^th ^Floor, Guy's Tower, Guy's Hospital, London, United Kingdom; 2 Department of Epidemiology and Public Health, Imperial College School of Medicine, London, United Kingdom

## Abstract

We performed variance components linkage analysis in nuclear families from the Framingham Heart Study on nine phenotypes derived from systolic blood pressure (SBP). The phenotypes were the maximum and mean SBP, and SBP at age 40, each analyzed either uncorrected, or corrected using two subsets of epidemiological/clinical factors. Evidence for linkage to chromosome 8p was detected with all phenotypes except the uncorrected maximum SBP, suggesting this region harbors a gene contributing to variation in SBP.

## Background

Linkage analysis of quantitative traits holds great promise for dissecting the genetic contribution of phenotypes that vary in the population. While only the extreme trait values may be of clinical relevance, the full distribution in the population provides additional power to identify genetic determinants. However, the variation in trait value will depend on the underlying genes, additional environmental factors, and other correlated phenotypes that may also be under genetic control. The underlying trait distribution after removing these factors may have higher power to detect linkage. However, removing variation due to factors which themselves have a genetic basis (e.g., body mass index in systolic blood pressure) may remove genetic variation due to common genes contributing to both phenotypes, or where the genes for the explanatory variable (weight) contribute a major proportion of the genetic variation in the major phenotype of interest (systolic blood pressure).

In this study, we analyzed nine phenotypes from the Genetic Analysis Workshop 13 Framingham Heart Study data, based on systolic blood pressure (SBP). We explore how the choice of underlying phenotype (maximum, mean, or age-matched SBP) and correction for other variables affects the results of variance components linkage analysis.

## Methods

Three baseline SBP response variables were used in the analysis: mean SBP, maximum SBP, and SBP at age 40 (to analyze early onset cases). Mean and maximum SBP were calculated for all study participants in Cohorts 1 and 2; no age limit was imposed and no minimum number of SBP readings were required for an individual to be included in the analysis. For SBP at age 40, the value from the first visit following a participant's fortieth birthday was used, or the final reading if the participant was under age 40 at their final study visit. Any participant who had no SBP recorded between the ages of 35 and 45 years was excluded from the age 40 analysis. It was assumed that all missing data occurred at random and would not bias results.

For each of the three response variables, three levels of correction were applied. As well as the unadjusted response (A), adjustments were made for epidemiological and clinical covariates (B) and epidemiological covariates only (C). Adjusted phenotypes were obtained from the residuals after fitting a linear model using SPLUS v5.1. COHORT was included in all adjusted phenotypes, otherwise only those covariates that were statistically significant at the 5% level using a forward model selection process were included. For adjustment B, covariates included in the variable selection process were age, sex, cigarettes per day (SMOKE), units of alcohol per day (DRINK), treatment for high blood pressure (TREATED), and body mass index (BMI). Adjustment C included all covariates except TREATED and BMI. For maximum SBP and SBP at age 40, the corresponding covariate measure was used. When analyzing mean SBP, the covariates SMOKE, DRINK and TREATED were defined as yes or no according to whether these factors has ever occurred. The resulting nine phenotypes will be referred to as MAXSBP, MEANSBP, and AGE40SBP with suffixes A, B and C referring to the three levels of correction (Table 1). All response variables were log-transformed prior to adjustment, since variance components analysis is sensitive to non-normality of trait data [1]. Phenotypes were summarized by calculating phenotypic and sib correlations (Tables 1 and 2).

Extended pedigrees were split into nuclear families due to the constraints of GENEHUNTER. In total, 294 nuclear families (242 extended pedigrees) from 330 original pedigrees contributed to the analyses, having two or more genotyped sibs with trait values for at least one of the phenotypes studied (Table 1). Quantitative trait multipoint linkage analysis was performed using GENEHUNTER 2 [2,3]. The variance components (VC) method was used; this method compares the maximum likelihood model of the mean trait value from estimated environmental, polygenic, and quantitative tract loci (QTL) VC, with the null model assuming no QTL. The likelihood ratio of these models can be expressed as a LOD score, which is assessed for significance using a standard chi-squared test. Male and female trait values were estimated separately within GENEHUNTER, and purely additive QTL effects were assumed with no dominance variance.

## Results

**Figure 1:**
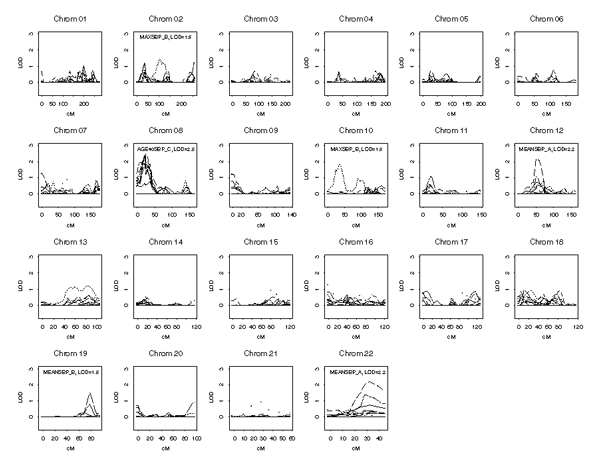
**Genome-wide linkage results from VC analysis. **Linked phenotype marked for regions with maximum LOD > 1.5.

**Figure 2:**
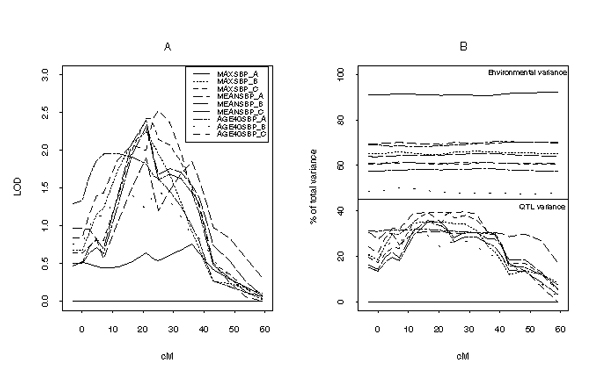
**Chromosome 8p linked region. **A, Multipoint LOD scores; B, Mean VC estimates.

Genome-wide linkage analysis results for each of the nine phenotypes are shown in Figure 1. The highest LOD score was obtained for chromosome 8 by AGE40SBP_C (maximum LOD = 2.5 at GATA23D06). All of the phenotypes except MAXSBP_A and AGE40SBP_B showed suggestive evidence for linkage (Figure 2), defined by a LOD score > 1.9 [2,4]), although no correction for multiple testing across phenotypes has been applied. The maximum LOD score for AGE40SBP_B was 1.5; weaker evidence for linkage using this phenotype may be due to the smaller sample size, as many individuals had no recorded DRINK variable at that SBP measurement. Only MAXSBP_A failed to reach a LOD score of 1.0. With the exception of the unadjusted maximum SBP (MAXSBP_A), 30% to 40% of the phenotypic variance is explained by a QTL in this region. Most of the variance (>90%) in the MAXSBP_A phenotype is explained by environmental factors.

Chromosomes 12 and 22 showed suggestive linkage with MEANSBP_A only; the highest LOD score in these regions from the remaining phenotypes was 1.1 (chromosome 12 AGE40SBP_A) and 1.4 (chromosome 22, AGE40SBP_A). In total, 14 regions across the genome attained a LOD score > 1.0. Of these, 11 included a phenotype adjusted for epidemiological and clinical covariates (B), compared with two unadjusted (A) and three adjusted for epidemiological covariates only (C). These results are consistent with the higher sib correlation observed for phenotypes B than C (Table 1). Each SBP measure (maximum, mean, age 40) contributed a similar number of high LOD scores.

## Discussion

We have shown suggestive linkage to chromosome 8 between 0 and 40 cM, with similar evidence for localization across most of the defined SBP phenotypes. No other region of the genome showed such high LOD scores, or such consistency across phenotypes. The chromosome 8 linkage results suggest that the gene(s) in this region contribute directly to SBP, and not to an intermediate phenotype which has been corrected for (e.g., BMI). All SBP-based phenotypes showed some evidence for linkage on chromosome 8 (LOD > 1.5), except the unadjusted MAXSBP. Further analysis of SBP corrected for individual epidemiological and clinical factors might identify the crucial factors to be included in the correction for SBP.

The maximum LOD score over the nine traits is 2.5. Although multiple testing has been performed, we note that the traits are not independent (Table 2) and that eight give suggestive LOD scores greater than 1.5. A Bonferroni correction would therefore be unnecessarily conservative. A multivariate phenotype analysis may be preferable, but is beyond the scope of this article.

Estimates of the environmental variance remain relatively constant as QTL location varies. However, it is noticeable that where high LOD scores are obtained, additive polygenic genetic variance is close to zero; that is, the putative QTL explains almost all of the additive genetic variance. This seems unlikely and leads us to consider possible deviations from model assumptions. The most obvious is failure of normality, which has been shown to lead to inflated type I error rates [1], but standard tests of normality showed no reason to doubt this assumption. One possible further check not implemented here would be the simulation-based test described in Iturria et al. [5].

The Framingham Heart Study allows many choices of summary measures from longitudinal data. We used maximum, mean, and age-specific SBP. The latter phenotype gave the highest LOD score (2.5 on chromosome 8p) and may reduce much of the age-dependent variation in SBP which is present in other unadjusted phenotypes. For example, the maximum SBP is treated similarly for study participants from age 20–30, or from age 40–70, although the distribution of SBP differs substantially in these age ranges. The linkage results suggest that modelling by age and epidemiological or clinical factors may increase the power to detect linkage to SBP.

## Figures and Tables

**Table 1:** Phenotypic summary

Phenotype	Final Model (models B and C include COHORT)	Sibling Correlation	Mean No. Genotyped & Phenotyped Individuals per Family	Total No. Families
MAXSBP_A	Unadjusted	0.554	3.17	294
MAXSBP_B	TREATED, BMI, SEX	0.245	3.17	293
MAXSBP_C	AGE, SEX	0.192	3.17	294
MEANSBP_A	Unadjusted	0.460	3.17	294
MEANSBP_B	TREATED, BMI, SEX, AGE, DRINK	0.304	3.17	294
MEANSBP_C	SEX, AGE, DRINK SMOKE	0.278	3.17	294
AGE40SBP_A	Unadjusted	0.317	3.17	294
AGE40SBP_B	TREATED, BMI, SEX, DRINK	0.225	2.26	228
AGE40SBP_C	SEX	0.189	3.00	269

**Table 2:** Phenotype correlations

Phenotype	MAXSBP			MEANSBP			AGE40SBP		
	
	A	B	C	A	B	C	A	B	C
MAXSBP_A	1	0.73	0.78	0.89	0.57	0.67	0.68	0.47	0.46
MAXSBP_B		1	0.93	0.73	0.78	0.79	0.60	0.57	0.52
MAXSBP_C			1	0.77	0.75	0.88	0.65	0.53	0.61
MEANSBP_A				1	0.76	0.84	0.84	0.65	0.68
MEANSBP_B					1	0.90	0.66	0.71	0.63
MEANSBP_C						1	0.73	0.69	0.75
AGE40SBP_A							1	0.88	0.94
AGE40SBP_B								1	0.92
AGE40SBP_C									1

## References

[B1] Allison DB, Neale MC, Zannolli R, Schork NJ, Amos CI, Blangero J (1999). Testing the robustness of the likelihood-ratio test in a variance-component quantitative-trait loci-mapping procedure. Am J Hum Genet.

[B2] Pratt SC, Daly MJ, Kruglyak L (2000). Exact multipoint quantitative-trait linkage analysis in pedigrees by variance components. Am J Hum Genet.

[B3] Kruglyak L, Daly MJ, Reeve-Daly MP, Lander ES (1996). Parametric and nonparametric linkage analysis: a unified multipoint approach. Am J Hum Genet.

[B4] Lander E, Kruglyak L (1995). Genetic dissection of complex traits: guidelines for interpreting and reporting linkage results. Nat Genet.

[B5] Iturria SJ, Williams JT, Almasy L, Dyer TD, Blangero J (1999). An empirical test of the significance of an observed quantitative trait locus effect that preserves additive genetic variation. Genet Epidemiol.

